# Detection of G-Quadruplex DNA Using Primer Extension as a Tool

**DOI:** 10.1371/journal.pone.0119722

**Published:** 2015-03-23

**Authors:** Rupa Kumari, Mridula Nambiar, Shaika Shanbagh, Sathees C. Raghavan

**Affiliations:** Department of Biochemistry, Indian Institute of Science, Bangalore, India; Tulane University Health Sciences Center, UNITED STATES

## Abstract

DNA sequence and structure play a key role in imparting fragility to different regions of the genome. Recent studies have shown that non-B DNA structures play a key role in causing genomic instability, apart from their physiological roles at telomeres and promoters. Structures such as G-quadruplexes, cruciforms, and triplexes have been implicated in making DNA susceptible to breakage, resulting in genomic rearrangements. Hence, techniques that aid in the easy identification of such non-B DNA motifs will prove to be very useful in determining factors responsible for genomic instability. In this study, we provide evidence for the use of primer extension as a sensitive and specific tool to detect such altered DNA structures. We have used the G-quadruplex motif, recently characterized at the *BCL2* major breakpoint region as a proof of principle to demonstrate the advantages of the technique. Our results show that pause sites corresponding to the non-B DNA are specific, since they are absent when the G-quadruplex motif is mutated and their positions change in tandem with that of the primers. The efficiency of primer extension pause sites varied according to the concentration of monovalant cations tested, which support G-quadruplex formation. Overall, our results demonstrate that primer extension is a strong *in vitro* tool to detect non-B DNA structures such as G-quadruplex on a plasmid DNA, which can be further adapted to identify non-B DNA structures, even at the genomic level.

## Introduction

DNA exists majorly in the B form except in certain regions of the genome such as telomeres, wherein G-quadruplex structures have been identified [1,2]. Certain altered DNA structures have also been reported in promoters of many genes such as c-*KIT*, c-*MYC* etc., which are implicated in the regulation of gene expression [3,4]. Apart from these classical roles, the importance of non-B DNA structures in imparting fragility to certain regions of the genome is increasingly becoming clear [5,6]. Recent studies have shown that non-B DNA structures such as G-quadruplexes and cruciforms make several regions of the genome fragile, by increasing their susceptibility to breakage [5,7–10]. Such DNA breaks can result in chromosomal translocations, gene duplications, inversions and deletions, which are characteristic features on the landscape of cancer cells [11].

Translocations are one of the major types of chromosomal aberrations observed in cancer, especially leukemia and lymphoma [11–13]. The t(14;18) translocation involving the *BCL2* gene on chromosome 18 and *IgH* loci on chromosome 14, is one of the most common and well-studied translocations in cancer [14]. Most of the breaks on the *BCL2* gene are clustered in a 150 bp major breakpoint region (mbr), which was previously shown to adopt a non-B DNA structure [7]. This structure was recently characterized as an intramolecular G-quadruplex by several biophysical and biochemical assays including circular dichroism, NMR, DMS protection, 3D-modelling and a reporter gene based assay within cells [8].

There are over 500 distinct chromosomal translocations identified in haematological malignancies so far. However, the mechanism of generation of majority of them are largely unknown. Therefore, it is imperative to design new methods to aid the identification of altered DNA structures, as they might hold the key in explaining the fragility of several such regions in the genome, especially those involved in chromosomal translocations.

Owing to their structures, non-B DNA can act as road blocks for passage of polymerases across DNA. This approach has been exploited in Taq polymerase arrest assays, which have been previously reported [8,15]. However, this assay has been conventionally performed on single-stranded DNA and uses oligomers of defined lengths, containing the structure forming motifs [8,15]. Therefore, it may not represent the physiological scenario wherein such structures are formed on double-stranded DNA and need unwinding before each round of replication. In the present study, we employ the G-quadruplex forming motif at the *BCL2* mbr as a proof of principle and propose the use of primer extension as a method to detect and study non-B DNA structures on plasmid DNA. Usage of Vent polymerase and multiple cycles of primer extensions improved the sensitivity of the assay. Further we show that pause sites corresponding to the G4 motif depend on the concentration of DNA template, presence of cations, which are known to support G-quadruplex formation and the position of the primers used. Importantly, the pause sites are absent when the motif is mutated, indicating the role of DNA structure.

## Materials and Methods

### Enzymes, chemicals, and reagents

Chemical reagents were obtained from Sigma Chemical Co. (USA) and SRL (India). DNA modifying enzymes were from New England Biolabs (USA) and Fermentas (USA). Radioisotope-labeled nucleotides were from BRIT (India).

### Oligomeric DNA

The oligomeric DNA used in the current study are BTM1 (C), 5’-ACAGACCCACCCAGAGCCCTCCTGCCCTCCTTC-3’; BTM2 (G), 5’-GAAGG AGGGCAGGAGGGCTCTGGGTGGGTCTGT-3’; BTM4 (G_6_), 5’-GAAGGAGGGCAGGAGGGCTCTATATAAATCTGT-3’; MN60, 5’-TCGACTCTAGAACAGACCCACCCAGAGCCCTCCTGCCCTCCTTCG-3’; MN61, 5’-GATCCGAAGGAGGGCAGGAGGGCTCTGGGTGGGTCTGTTCTAGAG-3’; MN62, 5’-TCGACTCTAGAACAGATTTATATAGAGCCCTCCTGCCCTCCTTCG-3’; MN63, 5’-GATCCGAAGAGGGCAGGAGGGCTCTATATAAATCTGTTCCTAGAG-3’; SCR21, 5’-AGTGCCACCTGACGTCTAAG-3’; SCR105, 5’-GGCGTATCACGAGGCCCTTTCGTC-3’; SCR83, 5’-CCCTGTTGACAATTAATCATCG-3’; and SCR190, 5’-TAGGTACATTGAGCAACTGAC-3’. The oligomers were gel purified as described [16].

### 5’ end-labeling of oligomers

The 5’ end-labeling of the oligomeric DNA was done using T4 polynucleotide kinase as described [16]. The labeled substrates were purified using Qiagen quick nucleotide removal kit and stored at -20°C until used.

### Plasmid construction

Double-stranded DNA containing the wild type sequence of the *BCL2* mbr G- quadruplex forming region was generated by annealing MN60/MN61 and the mutant which has two sets of guanines (6 guanines) substituted was made by annealing MN62/MN63. The duplex DNA, having BamHI and SalI overhangs, was phosphorylated and ligated to generate appropriate plasmids following double-digestion with BamHI and SalI [8]. The sequence of the insert was confirmed by DNA sequencing. The resulting plasmids were named as pMN7 (wild type) and the pMN8 (mutant), respectively.

### Linear amplification of the DNA to detect pause sites

Presence of replication blocks due to G-quadruplex structure formation was studied in the plasmids, pMN7 and pMN8, by primer extension. The reactions were carried out by mixing 100 ng (except when different concentrations of template DNA were used) of DNA sample in 1X Thermo polymerase buffer (10 mM KCl, 10 mM (NH_4_)_2_SO_4_), 20 mM Tris-HCl (pH 8.8), 4 mM MgSO_4_ and 0.1% Triton X-100), 4 mM MgSO_4_, 200 μM dNTPs, 0.1 nM end labeled oligomers and 1 U Vent (exo-) polymerase (New England Biolabs). Linear amplification primer extensions were carried out in a PCR machine (20 cycles) under the following conditions: 95°C for 3 min (1 cycle), 94°C for 45 sec, 56°C for 45 sec and 72°C for 45 sec (20 cycles) and final extension of 3 min at 72°C.

### Mapping of pause sites to G-quadruplex motif using differentially positioned primers

Occurrence of pause sites with respect to G-quadruplex forming motif was tested using multiple primers that can anneal at different positions, away from the G-quadruplex motif. The primers used were SCR21, SCR105 and SCR83, positioned 165, 111 and 67 bp, respectively, from the G4 motif. Primer extension was performed at annealing tempertures of 56, 58 and 60°C for 45 sec with 45 sec denatuaration and 45 sec extension, in case of both wild type and mutant plasmids. The reactions were terminated by adding dye containing formamide and the products were resolved on 8% denaturing polyacrylamide gel. The gels were dried and exposed to a screen and the signal was detected using PhosphorImager FLA9000 (Fuji, Japan).

### Primer extension in presence of different cations

Effect of different cations on the replication block during primer extension was studied by using the conditions described above, except that thermo polymerase buffer was supplemented by either 100 mM KCl, LiCl, NaCl or CaCl_2_. In the experiment to test the effect of increasing concentrations of KCl, the concentrations used were 0, 5, 10, 20, 50, 75, 100 and 125 mM.

### Dimethyl sulfate (DMS) protection assay

The 5’ radiolabelled oligomer, BTM2, containing the *BCL2* mbr G-quadruplex motif, was incubated in Tris-EDTA (TE) buffer either in the presence or absence of 100 mM KCl at 37°C for 1 h. DMS (1/200 dilution) was then added to the DNA and incubated for 15 min at room temperature. An equal volume of piperidine (10%) was added to each tube and incubated at 90°C for 30 min. The reaction was then diluted with water and vacuum dried. The pellet was washed several times and dried using a speedvac concentrator. The DMS treated products were then resolved on a 15% denaturing polyacrylamide gel, dried and visualized as described above.

### Circular Dichroism (CD)

*BCL2* mbr G-quadruplex motif containing plasmids, pMN7 (wild type) and pMN8 (mutant) were heat denatured for 10 min at 90°C in TE and renatured at 37°C for 1 h in the presence of 100 mM KCl. The samples incubated in TE alone served as the control. The circular dichroism spectra were recorded at 37°C from wavelengths of 220 nm to 320 nm. For each sample, 10 cycles of spectra were acquired; using a JASCO J-810 spectropolarimeter at a scan speed of 50 nm/min. Spectra for buffer alone or buffer with KCl was subtracted and plotted with zero-correction at 320 nm.

### Native gel analysis of intra and intermolecular G-quadruplex structure formation

The ability of *BCL2* mbr oligomers, BTM1 (C), BTM2 (G) and the mutant, BTM4 (G_6_) to form G-quadruplex structures was assayed as described with modifications [8,17]. The radiolabelled oligomers were either incubated in TE buffer (pH 8.0) or TE containing KCl (100 mM), NaCl (100 mM) or LiCl (100 mM) at 37°C for 1 h. The products were resolved on 12% native polyacrylamide gels containing respective salts (100 mM), and electrophoresed (100 V) in TBE buffer along with the salts, at room temperature. The gels were dried, images were captured and analyzed as mentioned above.

### Primer extension in presence of different cations

The presence of pause sites due to the formation of G-quadruplex structure at the *BCL2* mbr was studied on pMN7 by primer extension using the oligomer SCR105. The reactions were performed by mixing 100 ng of pMN7 in 1 X Themo polymerase buffer (New England Biolabs, USA), 4 mM MgSO_4_, 200 μM dNTPs, 0.1 nM end labelled SCR105, and 1 U Vent (exo-) polymerase. Besides 100 mM of either KCl, LiCl, NaCl or CaCl_2_ was also added to the reaction. Primer extension reaction was carried as described above (20 cycles) under following conditions: 95°C for 3 min (1 cycle), 95°C for 1 min, 58°C for 45 sec, 72°C for 45 sec (20 cycles) and the final extension at 72°C for 3 min. Formamide containing dye was used to terminate the reaction and products were resolved on an 8% denaturing polyacrylamide gel. The gels were dried and signals detected using a PhosphorImager (Fuji, Japan).

## Results and Discussion

### Primer extension can be used to detect non-B DNA structures *in vitro*

**Fig 1:**
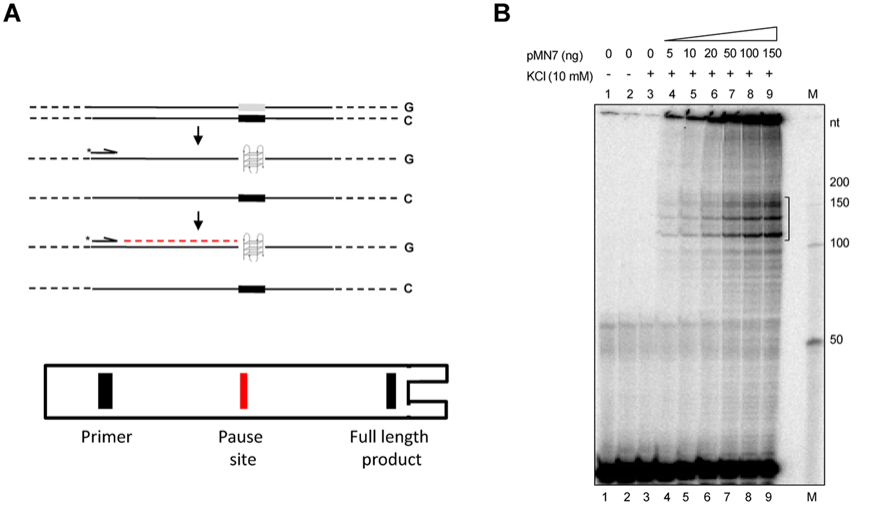
Primer extension assay to evaluate presence of non-B DNA structure. **A.** Schematic representation of the strategy of primer extension used for detecting pause sites due to altered DNA structures. Upon denaturation, the cloned sequence refolds into the non-B DNA (in this case a G-quadruplex) and acts as a replication block, preventing the extension of radiolabelled primer beyond the structure. This leads to the formation of a truncated product, which can be detected upon electrophoresis on a polyacrylamide gel. **B.** A denaturing polyacrylamide gel (8%) profile showing primer extension products using a radiolabelled primer SCR105 on increasing concentrations of the plasmid, pMN7 (0, 5, 10, 20, 50, 100 and 150 ng), containing the cloned wild type G-quadruplex forming motif, at an annealing temperature of 58°C. The pause site generated is marked with a square bracket. Lanes 1 and 2 are no DNA template controls without KCl and lane 3 is no DNA control with KCl. “M” is the radiolabeled 50 nt ladder and the molecular sizes are marked. In case of lanes 3–9, 10 mM KCl has been used. Wedge indicates the increasing concentration of pMN7.

Formation of non-B DNA structures like G-quadruplex, have been suggested to arrest physiological processes such as replication and transcription [18,19]. We tested whether such polymerase arrest due to formation of altered DNA structures can be detected using primer extension, when the region of interest is present on a double-stranded plasmid DNA (Fig. 1A). The plasmid containing the region of interest was denatured in a Thermo Cycler, annealed with a radiolabeled primer and allowed to extend for defined number of cycles. The primer can extend till the region where structure is formed, beyond which extension is impeded due to the presence of the non-B DNA. This will result in either the stalling of the polymerase or its falling off. This forms the truncated product, which can be observed on a denaturing polyacrylamide gel as a pause site (Fig. 1A). Since all the DNA molecules may not form the structure, significant amount of full length products can also be observed on the gel, which migrate the slowest (Fig. 1A). Unlike the Taq polymerase stop assay, where only a single cycle of polymerization is used, primer extension involves linear amplification due to which the signal strength is considerably improved. More importantly, we have used Vent (exo-) polymerase, instead of Taq DNA polymerase to improve the efficiency of the extension. In addition, the Vent (exo-) polymerase is highly thermostable and can resolve through highly GC-rich sequences substantially better than Taq polymerase, thereby reducing false positives [20,21].

We cloned the recently characterized wild type G-quadruplex forming motif from *BCL2* mbr, to generate pMN7 and used for the assay [8]. The primer extension assay was carried out using plasmid DNA (0, 5, 10, 20, 50, 100, 150 ng) and the primer SCR105, which binds around 100 nt away from the G-quadruplex motif. Results showed increasing intensity of pause sites near the position of the G-quadruplex motif, in a concentration dependent manner (Fig. 1B). The pause sites were spread over a distance of 50 nt spanning the G-quadruplex motif (from 100–150 nt). It is of importance to note that, apart from the pause sites, no other major bands were observed through the lanes, suggesting the specificity of primer extension in the detection of alterations in DNA structure on plasmid DNA.

### Pause sites move in tandem with the position of primers

**Fig 2:**
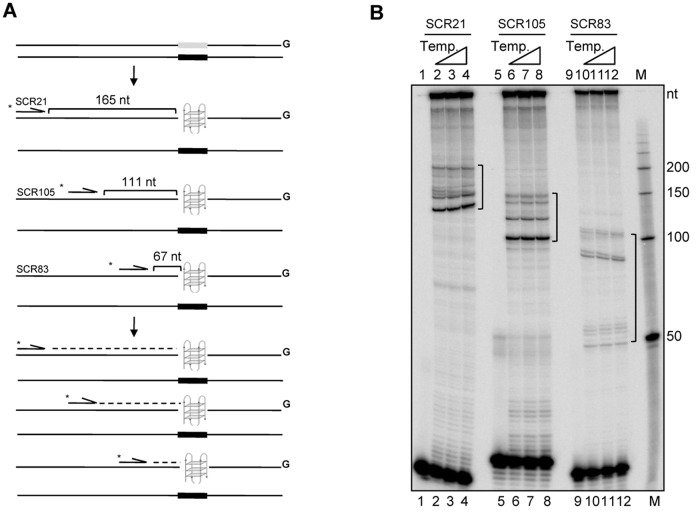
Primer extension assay using differentially positioned primers on a plasmid containing G-quadruplex forming motif. **A.** Schematic representation for the use of multiple primers which bind at different distances from the G-quadruplex forming motif. As a result, based on the distance between the primer and the motif, different sized products are expected. **B.** Polyacrylamide gel profile for primer extension reactions using primers, SCR21, SCR105 and SCR83, on the plasmid, pMN7, at different annealing temperatures. The annealing temperatures used were 56, 58 and 60°C. The pause sites generated as a result of replication blocks are marked in square brackets. Lanes 1, 5 and 9 are no DNA template controls. “M” is the radiolabeled 50 nt ladder and the molecular sizes are marked. The distance between primers SCR21, SCR105 and SCR83 and the G-quadruplex forming region is 165, 111 and 67 nt, respectively.

In order to further confirm the dependence of the pause sites on the cloned G4 DNA forming motif, we selected three different primers which would bind at different distances from the *BCL2* mbr G-quadruplex motif on the vector backbone (Fig. 2A). Results showed pause sites, in case of all the three primers at different temperatures tested (Fig. 2B). In case of primer SCR21, pause sites were observed at 150–200 nt region, for primer SCR105 it was between 100–150 nt and in case of SCR83, the pause was seen at 50–100 nt region (Fig. 2B). It needs to be pointed out that, like SCR21, the pause sites in case of extensions by SCR105 and SCR83 were specific in position. In case of SCR83, a pair of bands was observed at around 50 and 100 nt, which is identical in pattern to the pauses by other two primers but appears far apart due to the relatively closer position of primer and increased resolution of the bands at smaller molecular sizes.

### Conformation of G-quadruplex structure formation at the pause site

**Fig 3:**
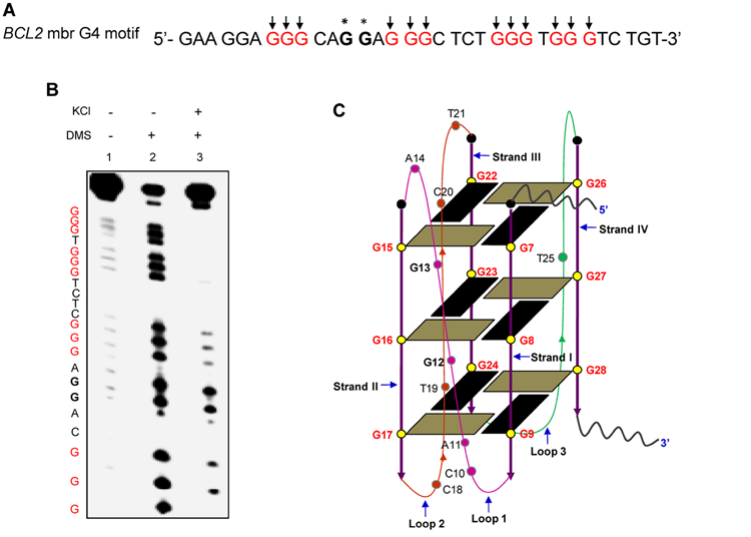
DMS protection assay to test G-quadruplex formation at *BCL2* mbr. **A.** Sequence of the G-rich strand at the *BCL2* mbr. The stretches of guanines which are protected from DMS methylation are in red and marked by arrows, while the two guanines which react to DMS within the motif are marked by asterisks. **B.** The gel profile showing DMS sensitivity at G-quadruplex motif in the *BCL2* mbr following chemical probing reaction. Lane 1, oligomeric DNA containing G-quadruplex motif treated with piperidine alone. Lane 2, DNA treated with DMS and piperidine following incubation in TE. Lane 3, DNA treated with DMS and piperidine after incubating in 100 mM KCl. **C**. A 2D model for the intramolecular G-quadruplex formed at the *BCL2* mbr based on the reactivity of guanines to DMS. The guanines involved in the quartet formation are in red.

DMS protection assay was used for determining the guanines involved in the formation of G-quadruplex structures. DMS methylates guanines at the N7 position, either in single-stranded or duplex DNA. If the guanines take part in Hoogsteen base pairing required for G-quadruplex formation, the N7 position will not be accessible for methylation and therefore, cannot react with DMS. Oligomeric DNA containing the *BCL2* mbr G-quadruplex motif was designed and DMS chemical probing was performed in the presence and absence of KCl (Fig. 3A). Results showed that all the guanines reacted equally to DMS in the absence of KCl (Fig. 3B, lane 2), while only two guanines (marked by asterisks) were strongly methylated by DMS in the presence of KCl (Fig. 3B, lane 3). Other guanines (marked in red and with arrows) showed complete or significant protection from methylation (Fig. 3B, lane 3). Thus our data provides evidence for occurrence of G-quadruplex structure at the *BCL2* mbr (Fig. 3C), which was also consistent with earlier report [8].

**Fig 4:**
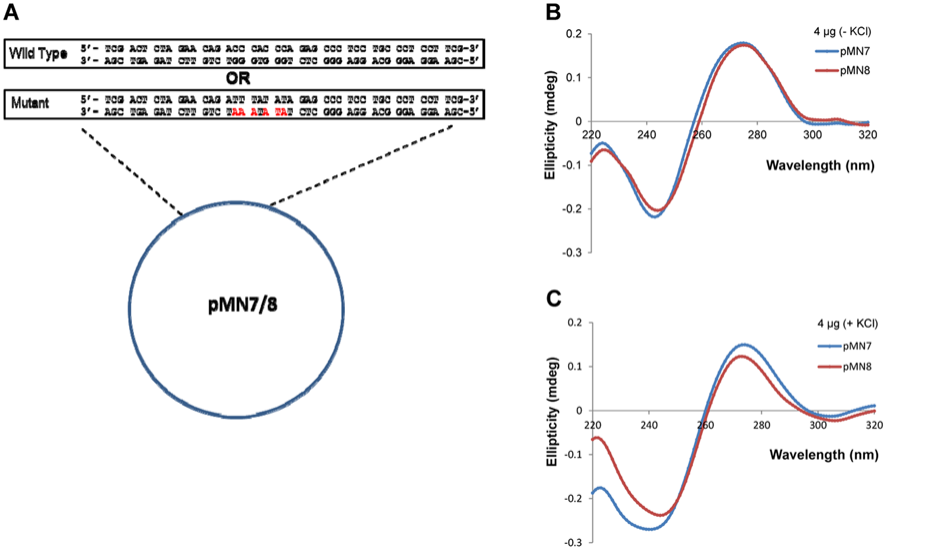
Evaluation of G-quadruplex formation at *BCL2* mbr, when present on a plasmid DNA. **A.** Plasmid constructs, pMN7 and pMN8, were prepared by cloning the wild type and the mutant oligomeric sequence containing G-quadruplex motif from *BCL2* mbr, respectively. **B-C.** CD spectra resulting from pMN7 and pMN8 in the absence (B) and presence (C) of KCl.

Besides, CD spectroscopic studies have been performed to identify the formation of G-quadruplex structure on a plasmid backbone. CD analysis was performed on pMN7 and pMN8 (Fig. 4A) in the absence or presence of KCl (100 mM). The characteristic spectra of B-DNA shows a positive long wavelength (peak) around 260–280 nm and a negative wavelength (dip) around 245 nm [22] while, parallel G-quadruplex formation could result in a positive peak at 260 nm and negative peak at 240 nm [22,23]. Results showed no difference in the pattern of the spectra between plasmid containing wild type and mutant G-quadruplex motifs in the absence of KCl (Fig. 4B). Interestingly, upon addition of KCl, pMN7 showed a distinct spectrum, characteristic of G-quadruplex as compared to its mutant (Fig. 4C). Thus, our results indicate the formation of a G-quadruplex DNA structure, in the context of plasmid DNA.

### Mutations to the G-quadruplex motif abolishes pause sites generated during primer extension

**Fig 5:**
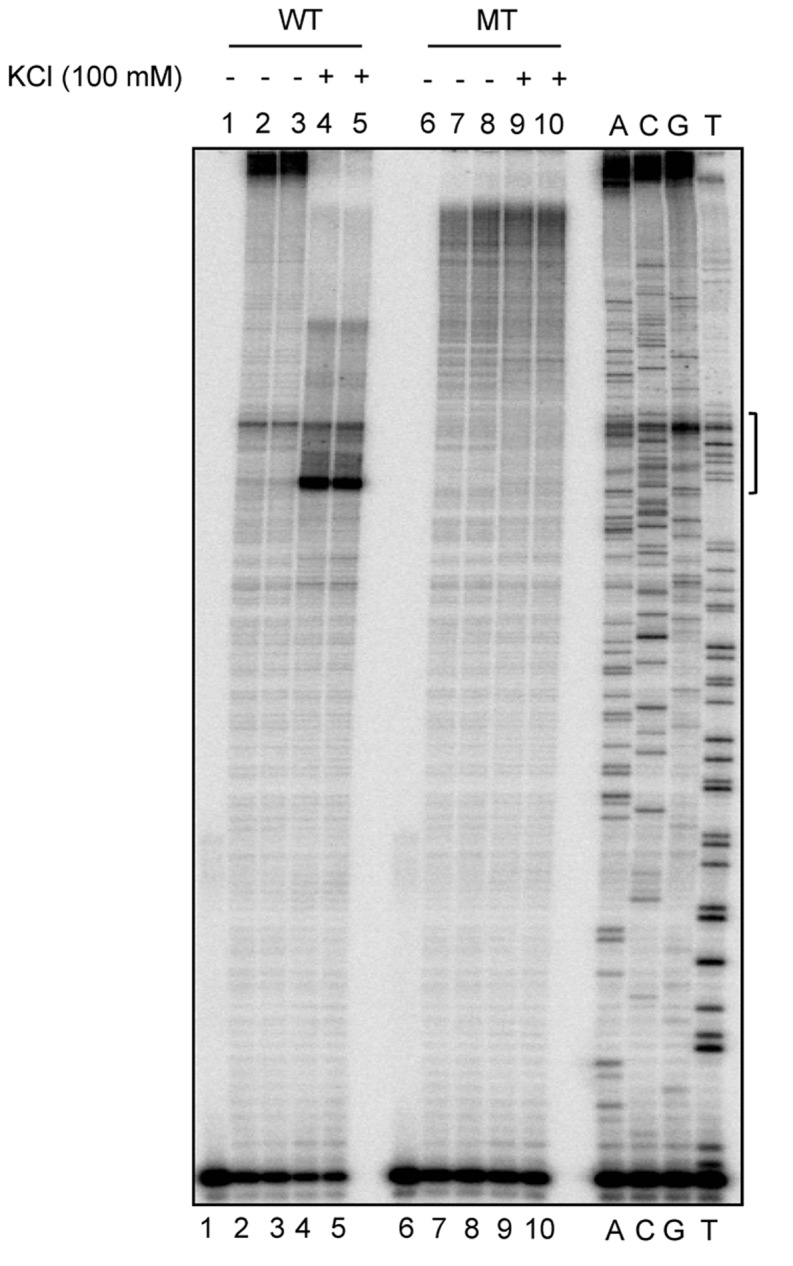
Primer extension on wild type G-quadruplex motif and its mutant. Primer extension using oligomer SCR105 on plasmids containing wild type (pMN7) or mutant (pMN8) G-quadruplex motif of *BCL2* mbr. In each case, Lane 1 and 6 are primer alone, lanes 2, 3, 7, 8 are primer extensions in the absence of KCl and lanes 4, 5, 9, 10 are in the presence of 100 mM KCl. The pause sites are marked with a square bracket. “WT” denotes wild type and “MT” denotes mutant plasmids. Sequencing ladder was prepared using pMN7 with primer SCR105 by Sanger’s chain termination method of sequencing. A, C, G and T denotes the corresponding ddNTP-mediated chain termination reaction.

**Fig 6:**
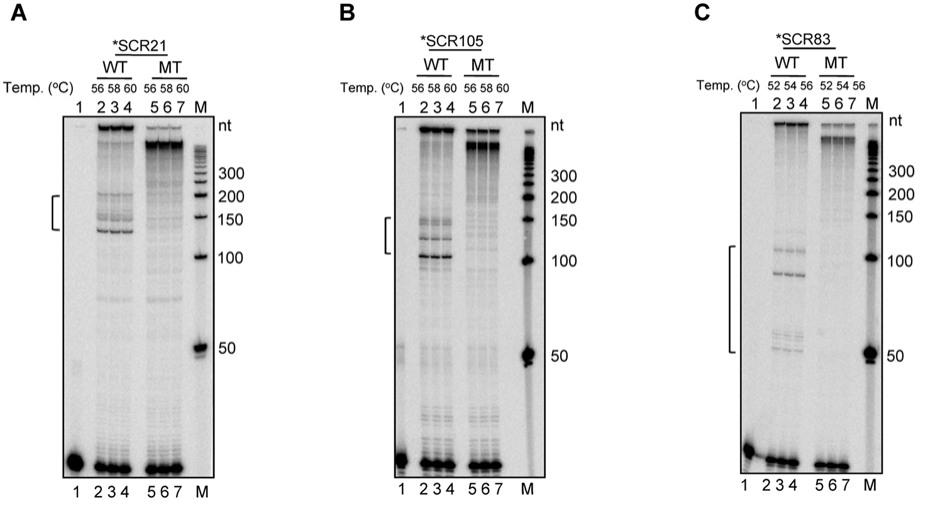
Primer extension on wild type and mutant G-quadruplex motif at *BCL2* mbr using multiple primers. **A-C.** Primer extension using oligomers, SCR21 (A), SCR105 (B) and SCR83 (C) for wild type and mutant plasmids. In each case, lane 1 is primer alone, lanes 2–4 are primer extensions at different temperatures as indicated. The pause sites generated as a result of replication blocks due to G-quadruplex formation are marked with square brackets. “WT” denotes wild type and “MT” denotes mutant plasmids. “M” is 50 nt ladder.

After establishing the ability to detect pause sites on plasmid DNA upon primer extension and demonstrating the ability of the sequence motif to fold into a G-quadruplex structure, we wanted to determine whether these replication blocks were indeed specific and dependent on the G-quadruplex motif. In order to test this, primer extension assay was performed on pMN8, the plasmid containing mutant of G-quadruplex motif (Fig. 4A). Upon primer extension, the mutant region showed a complete abrogation of the formation of pause sites even in the presence of KCl (100 mM), demonstrating that the pause site observed in the wild type plasmid was due to the presence of the G-quadruplex structure (Fig. 5). To further confirm the specificity of the pause sites, all the three primers used earlier, SCR21, SCR105 and SCR83, were used on the mutant plasmid as well. All the bands present in the wild type were absent in the mutant, except for the full extension products, which confirmed that each band seen in the former was due to a block generated by the non-B DNA structure (Fig. 6A-C). Interestingly, the full length products obtained from extension of the mutant sequence migrated faster than those from the wild type sequence, which was stuck in the well (Figs. 5, 6). Such a difference in the mobility of the full length products was unexpected and further studies are required to decipher the identity of these molecules.

### The G-quadruplex structure induced pause sites are dependent on monovalent cations

**Fig 7:**
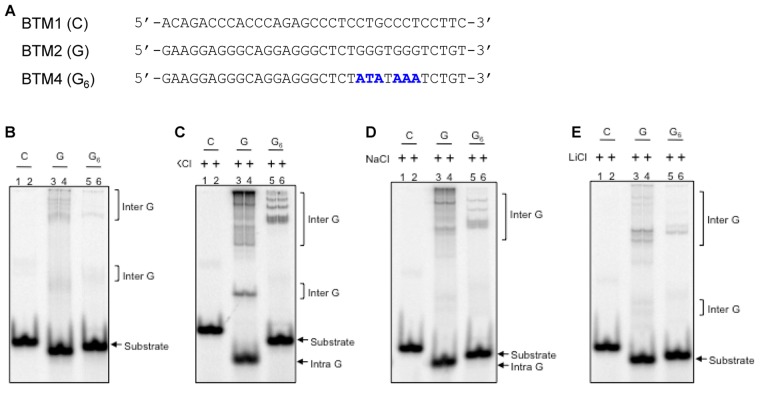
Evaluation of impact of different ions on G-quadruplex formation when *BCL2* mbr is present on oligomeric DNA substrates. **A.** Schematic representation of the oligomers used in the study. BTM1 (C) is the complementary sequence of G-quadruplex forming sequence and BTM2 (G) represents the G-quadruplex forming sequence of *BCL2* mbr. BTM4 (G_6_) represents the mutant form of BTM2 (G) where 6 Gs are mutated (indicated in blue color). **B-E.** Different oligomeric DNA, BTM1, BTM2 and BTM4 were incubated either in TE buffer (B), TE + KCl (C), TE + NaCl (D) or TE + LiCl (E) at 37°C for 1 h and resolved on a 12% native PAGE at room temperature. In all cases both the gel running buffer (TBE) and gel contained respective ions (100 mM). Formation of intermolecular species are marked by square brackets, while intramolecular G-quadruplexes and substrate DNA are indicated by arrows.

Previous studies suggest that monovalent cations such as K^+^ and Na^+^ favour the formation of G-quadruplex structures [8,10]. In order to test the effect of different ions on G-quadruplex structure formation at *BCL2* mbr, oligomers were designed containing either the G-quadruplex forming motif (BTM2), its complementary C-rich strand (BTM1) or its mutant (BTM4), where six Gs were mutated (Fig. 7A). The oligomers were labelled using [γ^32^P]ATP and incubated in the presence of different cations (100 mM KCl, NaCl or LiCl at 37°C for 1 h) and the products were resolved on a native PAGE as described in Methods. Results showed faster migrating species due to intramolecular G-quadruplex formation in the presence of KCl, when BTM2 was analysed on a gel containing KCl, when compared to control strands BTM1 and BTM4 (Fig. 7C). Interstingly, such a difference in the mobility was not observed in the absence of KCl (Fig. 7B). In presence of NaCl, although such a difference in the mobility was observed for BTM2, it was minimal (Fig. 7B-D). However, in the presence of LiCl, we could not observe any difference in the mobility and the migration was comparable to the condition wherein no ions were present (Fig. 7B, E). Interestingly, such a difference was not very prominent in the case of intermolcular G-quadruplex species (Fig. 7B-E). Thus, our results suggest that formation of G-quadruplex structure is favoured in the presence of KCl.

**Fig 8:**
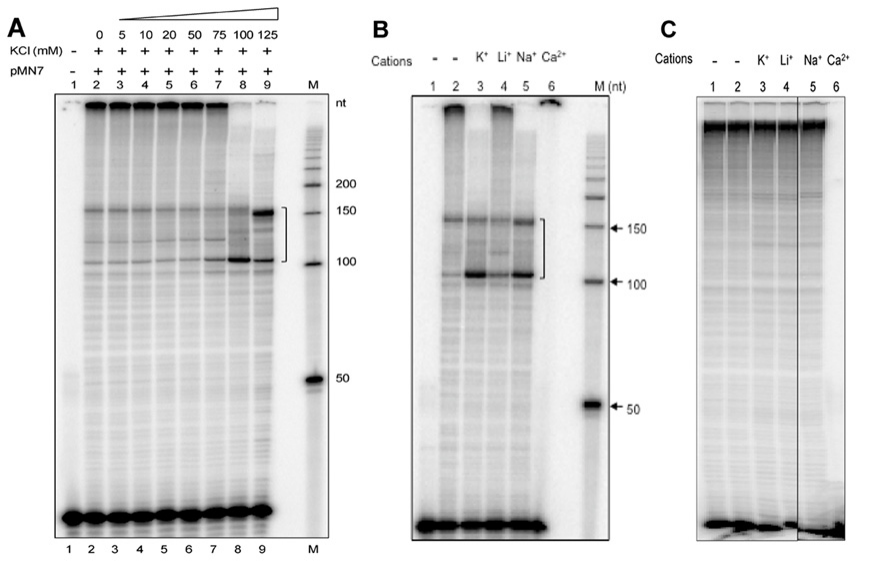
Primer extension through wild type and mutant G-quadruplex motif in the presence of different ions. **A.** Primer extension using oligomer SCR105 on pMN7 plasmid in the presence of increasing concentrations of KCl, (0, 5, 10, 20, 50, 75, 100 and 125 mM), which is indicated using a wedge. Lane 1 is no DNA control. **B, C.** Primer extension using oligomer SCR105 for wild type (**B**) and mutant (**C**) plasmids in the presence of either KCl, LiCl, NaCl or CaCl_2_ (100 mM). In panel B, lane 1 is no DNA control. The pause sites generated as a result of replication block due to G-quadruplex formation are marked by a square bracket. “M” is 50 nt ladder.

Based on the above result on shorter oligomers, we wondered whether the pause sites observed during primer extension were dependent on the presence of cations. Firstly, we tested the dependence of the pause sites on increasing concentrations of K^+^ ions. The primer extension reactions were carried out in presence of different concentrations of KCl (0, 5, 10, 20, 50, 75, 100 and 125 mM) (Fig. 8A). The results showed a concentration dependent increase in the formation of pause sites and the optimum was found to be 100 mM (Fig. 8A). Further, we carried out primer extension reactions in the presence of cations such as K^+^, Li^+^, Na^+^ and Ca^2+^. Results showed that strong pause sites were formed in the presence of KCl, and to a lesser extent when NaCl was present in case of the wild type sequence (Fig. 8B). Interestingly, in presence of LiCl, the formation of pause site was minimal. However, when the G-quadruplex motif was mutated, the pause sites were completely absent (Fig. 8C). Interestingly, CaCl_2_ did not support primer extension reaction, in both the wild type and mutant plasmids (Fig. 8B, C). Hence, the role of CaCl_2_ in supporting G-quadruplex formation cannot be commented upon. Overall, this result suggests that primer extension assay can be used to study the role of various cations in non-B DNA structure formation. Hence, this provides a simple method to test the favourable conditions for formation of various other non-B DNA structures *in vitro* as well.

Our results suggest that primer extension can be used as an efficient tool for detecting G-quadruplex DNA structures. In a recent study, we used primer extension to detect G-quadruplex DNA flanking the *HOX11* breakpoint region, involved in t(10;14) chromosomal translocation, which was further substantiated by several other biochemical and biophysical methods [10]. Another study used primer extension on S1 cleaved DNA by employing Taq polymerase to identify triplexes in the promoter region of a smooth muscle gene [24]. Primer extension reactions using human polymerases have also been reported to identify non-B DNA at common fragile sites [25]. It is of importance to note that both the latter studies use only a single cycle of extension, unlike the present study.

Biological role of non-B DNA structures is an active area of investigation. Besides chromosomal translocations associated with cancers, involvement of non-B DNA structures has also been suggested in various genetic diseases. In addition to G-quadruplex DNA structures, repetitive DNA can fold into structures like cruciform DNA, triplex DNA, left handed Z-DNA and slipped DNA conformations. Such altered DNA structures could result in aberrant DNA synthesis leading to repeat expansions and genomic rearrangements associated with neurodegenerative and genetic disorders such as Alzheimer’s and Huntington’s disease [6,26–32]. Thus, primer extension can become a useful tool to study DNA fragility in such diseases.

Although in principle, primer extension described here is similar to Taq polymerase stop assays, it is different in several ways. Conventionally, the templates used in Taq polymerase stop assays were single-stranded oligomeric DNA and used only a single cycle of amplification. Although it worked efficiently on simple oligomeric assay system, the major drawback was the low sensitivity, particularly when tested on longer DNA templates such as plasmid DNA. However, the current modified assay using increased number of cycles and Vent DNA polymerase helped in improving the sensitivity of the assay system significantly as described above. Similar technique based on primer extension has also been the fundamental basis for sequencing reactions and is successfully used to detect the 5’ ends of RNA [33, 34]. It has also been used previously to detect DNA breaks induced by endonucleases *in vitro* [7, 35].

In conclusion, this study reports primer extension as a strong *in vitro* tool for deciphering the presence of G-quadruplex motifs and other non-B DNA structures, using double-stranded plasmid DNA, by utilizing their property to act as blocks for polymerases during replication. It also provides an important and easy technique to test the role of various conditions such as presence of cations that can improve the efficiency of formation of such non-B DNA structures. This improved strategy can also be further developed to detect altered structures on genomic DNA.
